# On the person in personal health responsibility

**DOI:** 10.1186/s12910-022-00802-y

**Published:** 2022-06-25

**Authors:** Joar Røkke Fystro, Bjørn Hofmann, Eli Feiring

**Affiliations:** 1 Department of Health Management and Health Economics, University of Oslo, Oslo, Norway; 2 Department of Health Sciences, Norwegian University of Science and Technology, Gjøvik, Norway; 3 Centre for Medical Ethics, University of Oslo, Oslo, Norway

**Keywords:** Bioethics, Health priorities, Health responsibility, Insurance, Medical ethics, Moral responsibility, Person, Personal responsibility, Personhood, Legal responsibility

## Abstract

In this paper, we start by comparing the two agents, Ann and Bob, who are involved in two car crashes. Whereas Ann crashes her car through no fault of her own, Bob crashes as a result of reckless driving. Unlike Ann, Bob is held criminally responsible, and the insurance company refuses to cover the car’s damages. Nonetheless, Ann and Bob both receive emergency hospital treatment that a third party covers, regardless of any assessment of personal responsibility. What warrants such apparent exceptionalism with respect to personal responsibility in the healthcare context? We turn our attention to an understudied aspect of the debate on personal health responsibility, namely, the conceptualisation of the person in need of emergency hospital treatment. Drawing on the research of Joshua Knobe and Shaun Nichols, we propose that a context-dependent conceptualisation of the person may help explain a reluctance to ascribe responsibility to the individual for negative health outcomes.

## Introduction

Consider the following case. Ann is driving the speed limit using due care when she becomes aware of large rocks tumbling down the roadside hill towards her car. Ann makes a move to avoid the rockslide, loses control of the car and crashes into a deep ravine. She is badly hurt in the accident and driven to the nearest hospital for treatment. After investigating, the police conclude that it cannot be ruled out that the car crash was caused by Ann’s response to an unforeseeable situation, and Ann is not held legally responsible for the crash. Her auto insurance company pays for the damages to the car, and a third party covers the emergency hospital treatment.

Now, consider another case. Bob is driving far above the speed limit. Before an almost blind curve, he decides to pass the car in front of him. Halfway into the curve, he becomes aware of oncoming traffic. Bob makes a move to avoid the car in the other lane, loses control and crashes into a deep ravine. He is badly hurt in the accident and driven to the nearest hospital for treatment. Bob lives in a country (like most other countries) that treats emergencies equally, irrespective of how the need for hospital treatment arose and without any question about personal responsibility. A third party covers the resultant emergency hospital treatment. Bob is, however, held criminally responsible for his reckless driving. Unlike Ann, Bob seems to knowingly have done wrong, and he must pay a substantial monetary fine. He loses his driving licence and is sentenced to prison for a period. Moreover, Bob’s auto insurance company finds that Bob was driving recklessly and denies his claim to pay for the car’s damages.

In the present paper, we assume that both Ann and Bob have access to emergency hospital treatment covered by a third party, regardless of any assessment of personal responsibility. In contrast to what is typically the case in healthcare, responsibility practices are important features of criminal and actuarial justice. What warrants such apparent exceptionalism with respect to personal responsibility in the healthcare context? There are many potential answers to this question, and the relevance and possible role of personal health responsibility has been extensively debated in the literature [1–12]. We turn to an understudied aspect of this debate and ask if the conceptualisation of the person in need of emergency hospital treatment may contribute to explaining a seemingly widely held reluctance to hold people responsible for their health.

This paper proceeds as follows. We begin by outlining the notion of personal responsibility. We then give a brief overview of standard arguments against personal health responsibility. Finally and most importantly, we present and discuss a novel approach to personal health responsibility that explains how a contextual understanding of the bounds of the self is important to the way we assess health responsibility.

## Personal responsibility

We understand personal responsibility as the type that warrants blaming (or praising) a particular person for an action they performed or an outcome they have caused. The underlying premise of holding a person responsible is that the action or outcome in focus is causally attributed to that person [13, 14]. Furthermore, the person must be morally responsible for the action or outcome. Many philosophers have posited that moral responsibility requires an adequate degree of control when performing an action, which is sometimes understood as considering the person ‘could have done otherwise’ (the control condition). In addition, the person must be able to reasonably foresee—or be expected to have foreseen—the likely consequences of an action and its moral significance (the epistemic condition) [15–17].

It is beyond this paper’s scope to consider the perpetual discussion about the sort of control and type of knowledge that personal responsibility requires.^1^ Let us instead return to our cases to better grasp what we want to emphasise. Ann is causally responsible for crashing her car and for the resulting injuries. Still, many will share the mindset that Ann could not be blamed for the accident. After all, she drove carefully. She could not have foreseen the sudden rockslide nor the instinctive reaction that caused the car crash; thus, she could not be expected to have acted otherwise. Regarding the control aspect of moral responsibility, she will not be personally responsible for crashing her car.

Bob is also causally responsible for his car crash and the resulting injuries. However, contrary to Ann, his actions appear to meet both the control and epistemic conditions of moral responsibility. Whereas he did not intentionally crash his car, he could have acted otherwise, regarding both the speed limit violation and the hazardous overtaking attempt. Further, he knew—or was reasonably expected to know—that he was breaking the law and that his choices may have resulted in harm to himself and other people. Such behaviours carry considerable health risks, but they do not carry health benefits. Thus, the risks Bob took seem unreasonable. Accordingly, although both Ann and Bob are causally responsible for their car crashes, only Bob seems to be responsible in a moral sense.

Moral responsibility is, in most cases, regarded as a necessary condition for legal responsibility [18]. Because Bob was found morally responsible for his reckless driving and the subsequent car crash, he was held criminally responsible and was subject to punishment, such as loss of freedom, monetary fines and condemnation as a wrongdoer. In the actuarial context, insurance companies typically do not pay for damages when the insured is intoxicated or if they crashed as a result of reckless driving (as Bob does). Moreover, the insured may, in such cases, be forced to reimburse—wholly or in part—other parties if the accident caused other damages or injuries. But in the healthcare setting exemplified in our cases, Bob is granted treatment, like every other patient, irrespective of assessments of responsibility.

## Standard objections to personal health responsibility

In a universal healthcare system, the argument that one’s personal responsibility for one’s own behaviour makes it appropriate that one bear the medical and/or financial burdens that arise as the result may seem irrelevant. However, as most healthcare systems face difficulties with prioritisation, the idea that personal health responsibility may play a role in allocating scarce resources has gained traction in needs-based systems in recent years [5]. This idea may be normatively justified with reference to luck egalitarianism—a responsibility-sensitive theory of distributive justice—which claims that distributions are just if ‘[…] people’s comparative positions reflect nothing but their comparative exercises of responsibility’ [19]. Inequalities that are the result of voluntary choices do not give rise to redistributive claims on others.

This line of thinking has been debated [4, 20, 21]. Further, responsibility-sensitive policies, such as denial of treatment, assignment of lower priority or co-payments based on responsibility, are controversial in the healthcare context. Thus, philosophical, normative and practical arguments have been put forward against the use of such measures [5, 10, 22–24]. These arguments can be summarised as follows.

First, as alluded to, one argument is that healthcare should be allocated based on medical needs and not in order to ensure that everyone receives what they morally deserve (the health-needs objection). This argument may be justified in different ways. To some, health is a special good because it plays a central role in establishing the fair equality of opportunity in society [25]. To others, health is a special good because health-related pain and suffering are morally basic phenomena that trump other types of harm and liabilities [26, 27]. Because healthcare is one (of many) determinants of health, it should accordingly be distributed to those in need; thus, the ideal of responsibility-sensitive fairness simply does not apply to healthcare [25].

Other arguments rely on the assumption that although the allocation of healthcare in principle may be responsibility-sensitive, it seems difficult to determine the extent to which an individual is responsible for their health in meaningful ways. Lifestyle choices are not always well informed and may be embedded in disadvantageous social practises (the avoidability objection) [2, 5, 8, 22, 23, 28, 29]. A range of factors outside the individual’s control determine health, such as factors related to brute luck, neuropsychology, genetics and epigenetics, as well as factors related to socioeconomic status. This argument about exogenous contributing factors to negative health outcomes lies at the core of the causation objection, which relates to the problem of how we should determine the factors that lead to a particular health state [5, 22, 23, 30]. For example, Bob may be asked to bear responsibility for the car crash depending on the voluntariness of his choices, his opportunities to have acted otherwise and the impact of bad luck. He may be considered blameworthy for his hazardous driving, as he has no appropriate excuses that could diminish his culpability. Nevertheless, he is only partly blameworthy for the subsequent car crash and the injuries that followed. Although he was driving his car recklessly, the resultant car crash and injuries that followed were partially the result of bad luck. Notably, Bob could have driven recklessly without suffering a crash.

Moreover, responsibility-sensitive measures may imply harsh results (the harshness objection) [22, 23, 31–36]. For example, if a third party were to deny coverage of Bob’s medical treatment because the injuries were the result of his negligent behaviour, it could be argued that the outcome is too harsh. That is, a moment of imprudence wreaks economic havoc for the rest of his life. Worse, he may not receive (adequate) healthcare due to his inability to pay. Furthermore, ascribing personal responsibility requires intrusive investigations into people’s lives (the intrusiveness objection) [4, 22–24], possibly violating privacy while damaging self-respect and potentially failing to treat fellow citizens as moral equals [31, 36, 37]. Also, responsibility may be undermined by social factors, and the adoption of responsibility-sensitive measures may adversely affect the socially disadvantaged (the inequity objection) [23, 25].

## Conceptualisations of the person

The arguments against personal health responsibility have received considerable attention in literature. However, many arguments seem to apply equally to criminal and actuarial contexts.^2^ For example, responsibility may be undermined by social factors and ascribing responsibility can disproportionally affect those worse-off. Furthermore, determining the causality between actions and outcomes, as well as to which degree a person is morally responsible, appear to pose the same challenges to the different contexts in cases such as dangerous driving [38].

Although some arguments, such as those of harshness and health-needs objections, may offer reasons to treat questions about personal responsibility in healthcare differently than in other contexts, one aspect of the debate remains understudied. In the next sections, we draw attention to the causal condition required to ascribe personal health responsibility and focus on the conceptualisation of the person as a possible reason for the apparent exceptionalism regarding responsibility ascription in the healthcare context, inasmuch as the conceptualisation of the person seems to influence how we attribute causal responsibility. We use the notions of ‘person’ and ‘self’ interchangeably. We ask the following question: ‘Does the way we understand the bounds of the self matter with regard to how we assess health responsibility?’

According to Joshua Knobe and Shaun Nichols, there are three prominent approaches to the notion of the self [39]. First, there is the bodily conception, wherein the self is everything ‘from the skin in’. We are embodied organisms; thus, the conceptualisation of the self must relate to the body. The body is, so to speak, our interface with the world and a precondition of our experience and knowledge [40].

Second, the psychological conception treats the self as consisting of mental states, such as thoughts, desires, convictions and experiences. However, the body is merely a carrier of our wishes, preferences and interests. The parts of the body that are not associated with psychological processes are external to the self and are not regarded as constitutive of identity.

Third, there is the executive conception of the self. In this view, the individual is confronted not only with the body’s physical features, but also with their psychological states. Humans must deal with both features, meaning that the self is something further than the body and its psychology. The executive self makes decisions in light of the psychological states and decides, for instance, between conflicting desires, and may choose to perform an action despite overwhelming emotions.

**Fig. 1:**
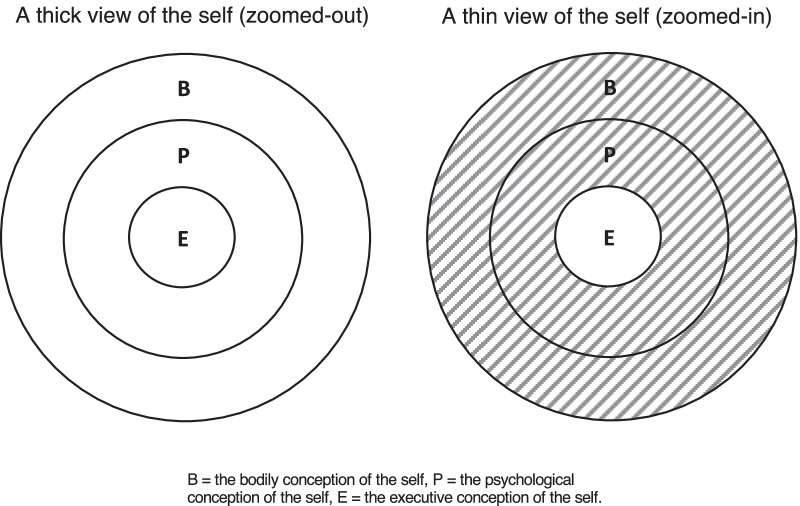
The three conceptions of the self and the two perspectives, i.e. zoomed-out and zoomed-in

Experimental findings [39] indicate that people harbour these concepts of self and one that is employed depends on the context, as we illustrate in Fig. 1. When people adopt a broad perspective and ‘zoom out’ to consider a behaviour in a wider context, perhaps involving many different people, they tend to adopt a bodily view of the acting individual’s self. From this perspective, the self includes the bodily, psychological and executive conceptions. On the other hand, when people ‘zoom in’ to consider a specific behaviour in isolation, they tend to see the physiological body and psychological states as falling outside the bounds of the self.

This contextual understanding of the self may be fruitful in helping one reflect on personal health responsibility as well. After all, it is necessary that the negative health outcome be causally attributed to the person; otherwise, it irrevocably undermines a personal health responsibility. Consider the following thought experiments inspired by Knobe and Nichols [39]:

- *Zoomed-in Case A (choice-cause)*
  Suppose Bob decides to pass the car in front of him. He speeds up, loses control of the car and crashes.
  Would you agree or disagree with the following statement?
  - Bob caused the car accident.
- *Zoomed-in Case B (emotion-cause)*
  Suppose Bob is driving when he is distracted by a wasp in his car. To avoid being stung, he makes a sudden move, loses control of the car and crashes.
  Would you agree or disagree with the following statement?
  - Bob caused the car accident.

From previous studies [39], we can expect that people would be willing to say that Bob caused the outcome in choice-cause Case A, but would be more reluctant to agree with the statement in emotion-cause Case B (and rather think that ‘Bob’s distraction caused him to make a bad move’). Thus, we would expect people to adopt a thin executive understanding of the self when discussing responsibility in these cases. Consider, then, the following:

- *Zoomed-out Case C (choice-cause)*
  Suppose Bob decides to pass the car in front of him. He speeds up, becomes aware of the oncoming traffic, makes a move to avoid the cars in the other lane, loses control and crashes. Several cars are involved in the crash. Many people are injured and require emergency hospital treatment.
  Would you agree or disagree with the following statement?
  - Bob caused the car accident.
- *Zoomed-out Case D (emotion-cause)*
  Suppose Bob is driving when he is distracted by a wasp in his car. To avoid being stung, he makes a sudden move and the car swerves into the other lane with oncoming traffic. He loses control and crashes. Several cars are involved in the crash. Many are injured and require emergency hospital treatment.
  Would you agree or disagree with the following statement?
  - Bob caused the car accident.

The research of Knobe and Nichols suggests that in zoomed-out conditions, people tend to ascribe causal responsibility regardless of the choice–emotion division [39]. When considering the broader context, people tend to include the body and mental states as part of the acting self, rather than thinking of the self as a thin executive. What about considerations regarding health responsibility? We suppose that people would be more willing to ascribe responsibility for health outcomes in the zoomed-out Cases C and D, i.e. when considering priority-setting dilemmas in which different patients or groups compete with each other for resources, insofar that they would be more willing to state that Bob caused the car crash. Intuitions may vary, however, in zoomed-in Cases A and B, depending on whether a health state is seen as a result of choice (A) or various mental states (B) (see Table 1).

**Table 1:** Comparison of health responsibility in zoomed-in and zoomed-out cases with respect to cause

	Zoomed-in	Zoomed-out
Choice-cause	Reckless driving due to choice results in injuries *Health responsibility (more likely) ascribed because of causal attribution to the self*	Competing healthcare needs Reckless driving due to choice results in injuries *Health responsibility (more likely) ascribed because of causal attribution to the self*
Emotion-cause	Reckless driving due to distraction results in injuries *Health responsibility ****not**** ascribed because of ****no**** causal attribution to the self*	Competing healthcare needs Reckless driving due to distraction results in injuries *Health responsibility (more likely) ascribed because of causal attribution to the self*

Moreover, we may speculate that there are differences depending on whether we consider the criminal, actuarial or healthcare contexts. Consider the following cases in which Bob, as in the introduction, appears to fulfil the control and epistemic conditions of moral responsibility:

- *Criminal/actuarial case*
  Suppose Bob decides to pass the car in front of him. Bob knows that he is an inexperienced driver, but he has purchased car insurance. He is well aware of the speed limit, but he still speeds up. Halfway into a nearly blind curve, he becomes aware of oncoming traffic. He makes a move to avoid the car in the other lane, loses control of the car and crashes.
  Would you agree or disagree with the following statement?
  - Bob caused the car crash.
- *Healthcare case*
  Suppose Bob decides to pass the car in front of him. Bob knows that he is an inexperienced driver and is well aware of the speed limit, but he still speeds up. Halfway into a nearly blind curve, he becomes aware of oncoming traffic. He makes a move to avoid the car in the other lane, loses control of the car and crashes. Bob is severely injured.
  Would whether you agree or disagree with the following statement?
  - Bob caused his severe injuries.

We expect that people would think Bob is personally responsible for the car crash if they were to assess responsibility from the criminal or actuarial contexts. However, we hypothesise that when assessing responsibility in the healthcare context, people would zoom-in closely and consider the many mental and physical factors that played roles in the decision to drive too fast. Hence, it will not be obvious that Bob was the cause. When we consider the precise processes that led up to Bob’s decision to drive faster than the speed limit and pass the car in front of him, we may assume that Bob’s desire for speed, his risk-seeking character, his lack of ability to understand the potential consequences and his lack of future-orientation are all factors that fall outside the bounds of the self. These are all factors that Bob must confront when making a decision. Thus, he seems not to be responsible, or at least to be responsible to a lesser degree, for driving too fast, the car crash and his subsequent need for healthcare.

However, the theory of context-dependent intuitions about the self cannot explain why the different contexts discussed here, i.e., the criminal, actuarial and healthcare, would matter. To get a sense of how we may approach a wider contextual understanding of the self, it may help to consider the idea that responsibility practices are social practices. Manuel Vargas’ formulation illustrates this point nicely: ‘Whether someone is a felon, crass, or deserving of ejection from a game is a matter both of the features of the individual agent and the action, but also of the operative social practice in which those agents and their actions have meaning’ [41]. Different spheres have different responsibility practices, and how we understand responsibility is best seen as the answers to questions about the rules that are internal to a specific practice. Against this backdrop, we suggest that people are more prone to regard choices as circumstantial when considering negative health outcomes than when considering outcomes in the criminal and actuarial spheres.

The mechanism that can contribute to explaining a tendency to zoom-in in the healthcare context may be the following. When assessing the need for healthcare, people adopt an individual viewpoint. We are not used to thinking of healthcare as a scarce resource that must be distributed among patients or groups; we tend to consider a particular patient’s healthcare needs in isolation. In the criminal and actuarial contexts, people adopt broader viewpoints on the decision. Reckless driving is unlawful because of the possibility of severe consequences for both the driver and other people. Thus, the drivers must take responsibility for their driving. Moreover, when assessing actuarial responsibility, we are well aware of the problem of ‘moral hazard’, i.e. that people take higher risks knowing that an insurer will pay the associated cost, thus destabilising the (car) insurance market. When assessing responsibility for healthcare needs, on the other hand, there may be an inclination to zoom-in and consider the individual’s consequences in isolation. Thus, what matters most to our assessment of responsibility in this context is the case details—not so much the broader picture. We may regard feelings of happiness, the lack of driving experience and a supposedly immature brain as aspects of the choice-situation that Bob faces, not as being a part of Bob. The physical features of Bob’s body and his psychological states seem external to his executive self. Considering this perspective, Bob’s severe injuries seem to be, to a lesser degree, his own fault; they manifest to a lesser degree as causally attributed to *him*. In other words, the broader context in which we assess responsibility affects our very assessments.

## Limitations

This proposal has several limitations. First, the hypothesis that we are more prone to adopt an individual perspective in the healthcare context, and thus more reluctant to ascribe health responsibility because we zoom-in and adopt a thin executive understanding of the self, is an empirical claim of human psychology and requires further research. However, medical ethics have a long tradition of being person-oriented. The doctor–person encounter has been the basic scenario for medical ethics, and basic ethical principles, such as autonomy, non-maleficence and beneficence, are person-oriented. But the recent SARS-CoV-2 pandemic (as well as other collective threats, such as climate change [42]) may alter this zoomed-in perspective. Thus, our perceptions of our personal responsibility for health may change.

Second, if the proposed cognitive mechanism and the way it influences the assessment of responsibility turn out to be valid, it may contribute solely to explaining the seeming reluctance to ascribe personal responsibility for negative health outcomes. The mechanism cannot substantiate whether we should account for personal responsibility in healthcare.

Third, responsibility is not a single, unitary and generic concept. Nicole A. Vincent has discussed how criminal law responsibility is a syndrome of at least six different concepts [43]. A thorough examination of the differences between responsibility concepts in different spheres is warranted.

## Conclusion

Although responsibility practices are an important feature of criminal and actuarial justice, personal responsibility in the healthcare context is far more controversial. Many believe that an individual can be personally responsible for their beliefs, intentions and actions, as well as the outcomes thereof. However, they may still argue that there are reasons why responsibility attributions should not play a role in healthcare. This may very well be explained by the standard objections to personal health responsibility and the fact that the moral justifications of healthcare are inherently different from those of criminal and actuarial justice. Nonetheless, in this paper, we have proposed that a context-dependent conception of the person may contribute to explaining the reluctance to ascribe personal responsibility for negative health outcomes.

## Data Availability

Data sharing is not applicable to this article as no datasets were generated or analysed during the current study.

## Footnotes

1 Although moral responsibility (and free will) skeptics may assert that we are never in control over our actions, and thus are never morally responsible, it does not explain the apparent healthcare exceptionalism.

2 We are thankful to an anonymous reviewer for bringing this point to our attention.
